# Novel enzyme-polymer conjugates for biotechnological applications

**DOI:** 10.7717/peerj.27

**Published:** 2013-02-12

**Authors:** Oscar Romero, Cintia W. Rivero, Jose M. Guisan, Jose M. Palomo

**Affiliations:** Departamento de Biocatálisis, Instituto de Catálisis (CSIC), Spain

**Keywords:** Mutagenesis, Lipase, Tailor-made polymer, Chemical modification, Biotransformations, Drugs precursors, Biocatalyst, Nucleosides, Protein chemistry

## Abstract

In the present research, a rapid, simple and efficient chemoselective method for the site-directed incorporation of tailor-made polymers into protein to create biocatalysts with excellent properties for pharmaceutical industrial purpose has been performed. First we focused on the protein engineering of the *Geobacillus thermocatenulatus* lipase 2 (BTL2) to replace the two cysteines (Cys65, Cys296) in the wild type enzyme (BTL-WT) by two serines. Then, by similar mode, a unique cysteine was introduced in the lid area of the protein. For the site-directed polymer incorporation, a set of different tailor-made thiol-ionic-polymers were synthesized and the protein cysteine was previously activated with 2,2-dithiodipyridine (2-PDS) to allow the disulfide exchange. The protected BTL variants were specifically modified with the different polymers in excellent yields, creating a small library of new biocatalysts. Different and important changes in the catalytic properties, possible caused by structural changes in the lid region, were observed. The different modified biocatalysts were tested in the synthesis of intermediates of antiviral and antitumor drugs, like nucleoside analogues and derivatives of phenylglutaric acid. In the hydrolysis of per-acetylated thymidine, the best biocatalyst was the BTL*-193-DextCOOH , where the activity was increased in 3-fold and the regioselectivity was improved, reaching a yield of 92% of 3’-*O*-acetyl-thymidine. In the case of the asymmetric hydrolysis of dimethyl phenylglutarate, the best result was found with BTL*-193-DextNH2-6000, where the enzyme activity was increased more than 5-fold and the enantiomeric excess was >99%.

## Introduction

**Figure 1:**
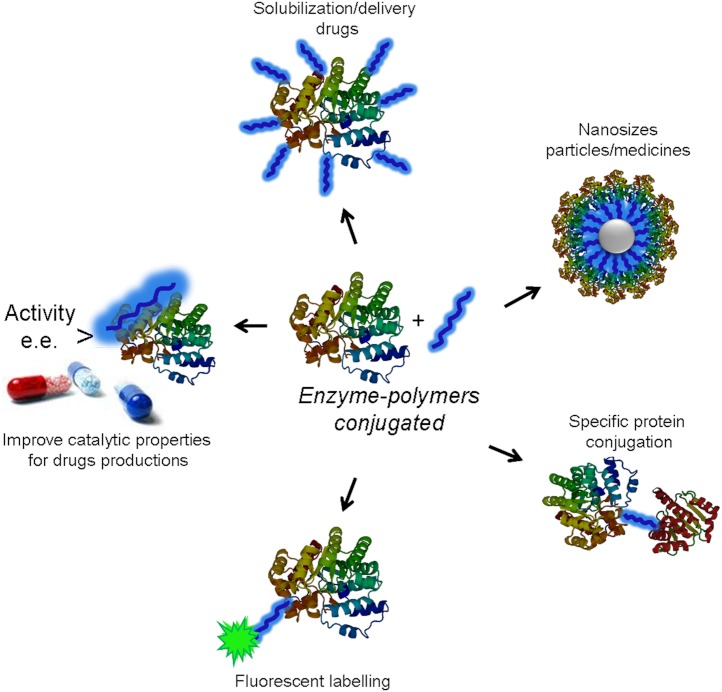
General scheme of the different applications of the site-directed modification of enzymes by tailor-made polymers.

Modification of protein with polymers is a widely employed technique for applications in medicine, biotechnology and nanotechnology (Gauthier & Klok, 2010; Haag & Kratz, 2006). The incorporation of polymers into proteins improves their stability, solubility and biocompatibility (Fig. 1). Additionally, this kind of modification can also alter the catalytic properties of enzymes for the creation of novel active and selective biocatalysts (Díaz-Rodríguez & Davis, 2011; Romero et al., 2012).

A variety of available methods for protein modification has been reported (Hermanson, 2008). However, modification of the nucleophilic thiol of a unique cysteine is a convenient and widely employed strategy to obtain a site-selective bioconjugation (Chalker et al., 2009). A unique cysteine can be easily introduced at virtually any position within a protein structure by site-directed mutagenesis and then selectively modified using disulfide compounds, for example (Romero et al., 2012).

The lipases are currently the most used enzyme in biocatalysis and organic chemistry (Drauz, Groger & May, 2012). In particular, the use of lipase to catalyze the asymmetric reactions has attracted great interest, due to them being able to accept a broad range of substrates with good activities, regio- and enantioselectivity (Faber, 2011). Moreover, in some cases, these good properties of lipases are insufficient, being the incorporation of polymers a useful and single alternative to improve them (Gutarra et al., 2011).

On the other hand, lipases are protein with a complex catalytic mechanism. The lipases have a polypeptide chain, called lid, that cover its catalytic site from the medium, but in the presence of an hydrophobic surface (hydrophobic support, oil drops, detergents, etc.) this lid moves leaving the active site exposed to the medium, so called interfacial activation (Verger, 1997). One special and interesting case is the lipase from *Geobacillus thermocatenulatus* (BTL2). This enzyme is the first crystallized lipase with two lids, which implies a more complex catalytic mechanism (Carrasco-López et al., 2009). Taking advantage of this tricky lid, the idea was to introduce a unique cysteine at different positions of the lid and modify with thiolated-ionic polymers afterwards. Using different mono-cysteine mutants and different polymers we have created a small library of different polymers-protein conjugates.

**Figure 2:**
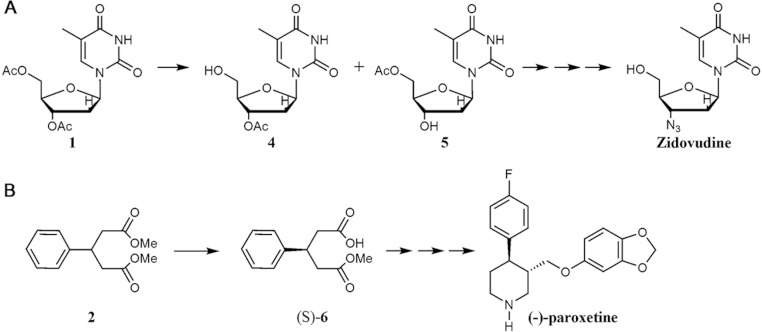
Biotransformations used in this work for synthesis of drug precursors. (A) Regioselective monodeprotection of per-*O*-acetylated thymidine; (B) Desymmetrization of dimethylphenylglutarate.

This small library of biocatalysts was tested in two biotransformations: regioselective deprotection of per-*O*-acetylated thymidine (**1**) and the desymmetrization of dimethylphenylglutaric acid diester (**2**). These are two important biotransformations with fascinating biotechnology application. For example, nucleoside analogues have attracted intense interest in medicine, where they are used as antitumor and antiviral agents (Galmarini, Mackey & Dumontet, 2002; Herdewijn, 2008). In the past 25 years, several nucleoside analog reverse-transcriptase inhibitors have been licensed and used in treatment of HIV/AIDS, for example Zidovudine (Esté & Cihlar, 2010) (Fig. 2). In the case of the enzymatic asymmetric hydrolysis of prochiral compounds this is a simple and effective route for the production of chiral compounds (García-Urdiales, Alfonso & Gotor, 2005), such as the diesters of phenylglutaric acid (Cabrera & Palomo, 2011). There are a high number of pharmaceutically important compounds containing a 3-arylglutaric building block, as (-)-paroxetine, (R)-baclofen, etc. (Fig. 2).

## Materials and Methods

### Materials

Sepharose^®^ 4BCL activated with cyanogen bromide (CNBr) were from GE Healthcare (Uppsala, Sweden). Dextran (Mr 1500, 6000), polyethyleneimine (PEI) (Mr 1500 kDa), 2-dipyridyldisulfide (2-PDS), diethyl-p-nitrophenylphosphate (D-pNP), dithiothreitol (DTT), L-aspartic acid, sodium borohydride, Triton^®^ X-100, dimethylsulfoxide (DMSO), 1-ethyl-3-(3-dimethylaminopropyl)-carbodiimide (EDC), hydroxylamine hydrochloride, n-hydroxysuccinimide (NHS), cysteine hydrochloride, ethylenediamine (EDA), thymidine, Dimethyl 3- phenylglutarate (**2**), p-nitrophenylbutyrate (pNPB) (**3**) and *N*-Succinimidyl-*S*-acetylthioacetate were from Sigma. Dextran-aldehyde, aspartic-dextran and amines-dextran prepared as previously described (Fuentes et al., 2004; Guisán et al., 1997). 3’,5’-di-*O*-acetylthymidine (**1**) was synthetized as previously described (Bavaro et al., 2009). Other reagents and solvents used were of analytical or HPLC grade.

### Site-directed mutagenesis of BTL

*B. thermocatenulatus* lipase (Genkbank number X95309) mutants were prepared as previously described (Romero et al., 2012). Briefly, all site-directed mutagenesis experiments were carried out by PCR using mutagenic primers (See ESI, Table S1). To introduce the amino acid change, the corresponding pair of primers was used as homologous primer pair in a PCR reaction using a specific plasmid as a template and Prime Start HS Takara DNA polymerase. The product of the PCR was digested with endonuclease DpnI that exclusively restricts methylated DNA (Schmidt-Dannert et al., 1996) *E. coli* DH10B cells were transformed directly with the digested product. The plasmid with mutated BTL were identified by sequencing and then transformed into *E. coli* BL21 (DE3) cells to express the corresponding proteins. Firstly, C65S was created, and the resulting plasmid was used as a template to create the double mutant C65S/C296S-BTL. This plasmid was used as a template to construct additional mutations (A193C, L230C) using different mutagenic primer (Romero et al., 2012).

### Cloning, expression, purification and immobilization of BTL variants

The gene corresponding to the mature lipase from *G. thermocatenulatus* BTL was cloned into pT1 expression vector, as previously described (Schmidt-Dannert et al., 1996). Cells carrying the recombinant plasmid pT1BTL2 were grown at 30 °C and over expression were induced by raising the temperature to 42 °C for 20 h. The enzyme was purified from *E. coli* crude extract by interfacial adsorption on Octyl-Sepharose as previously described (Fernandez-Lorente et al., 2008). The lipase was desorbed from the support adding 20 mL of 25 mM phosphate buffer pH 7 with 0.5% Triton X-100 (v/v) per gram of support. After that, the lipase was immobilized on CNBr-activated Sepharose at pH 7 in 25 mM sodium phosphate buffer for 1 h at 25 °C ( > 95% immobilization yields) with a final loading of 5 mg_lip_/g_cat_ .

### Enzymatic hydrolysis of pNPB

The activities of the soluble lipases (for the immobilization process) and their immobilized preparations were analyzed spectrophotometrically measuring the increment in absorbance at 348 nm produced by the release of p-nitrophenol (pNPOH) (∈ = 5.150 M^−1^ cm^−1^) in the hydrolysis of 0.4 mM pNPB in 25 mM sodium phosphate at pH 7 and 25 °C. To initialize the reaction, 0.05–0.2 mL of lipase solution or suspension was added to 2.5 mL of substrate solution. Enzymatic activity is given as micromole of hydrolyzed pNPB per minute per milligram of enzyme (IU) under the conditions described above.

### Preparation of different thiol-polymers

#### Preparation of thiol-aspartic-dextran (DextCOOH)

**Figure 3:**
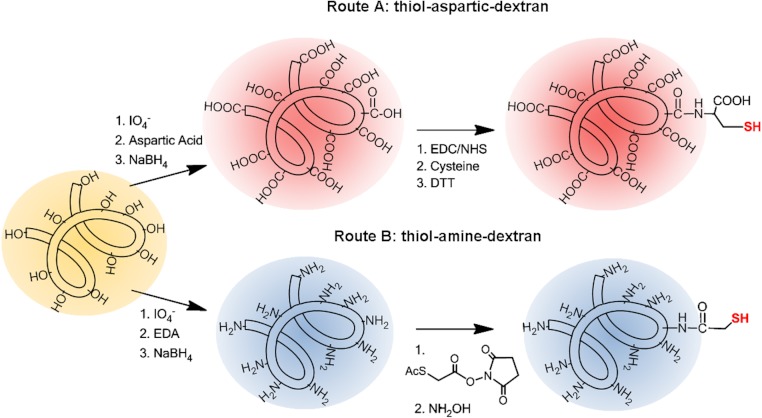
Synthesis of tailor-made thiol-dextran polymers.

To a 100 mL of dextran-aspartic polymer solution (10 mg/mL) Mw. 1.500 in 5 mM sodium acetate buffer at pH 5.0 were added 16 and 9.6 mg (0.125 eq) of EDC and NHS, respectively. Similar to before, to a 100 mL of dextran-aspartic polymer solution (10 mg/mL) Mw. 6000 in 5 mM sodium acetate buffer at pH 5.0 were added 1.0 and 0.6 mg (0.0313 eq) of EDC and NHS, respectively. For both polymers, the reaction was maintained for 2 h at 25 °C. After that the mixture was then dialyzed 3 times against distilled water (300 volumes). Then, the pH of polymer solution was set at 7.5 in 5 mM sodium phosphate buffer pH 7.5. Subsequently, 420 µL (0.0625 eq) or 26 µL (0.0156 eq) of a 100 mM cysteine solution were added to polymers of Mw 1.500 and 6.000, respectively. The reaction was maintained for 2 h at 25 °C and finally the mixture was extensively dialyzed (See Fig. 3).

#### Preparation of thiol-amine-dextran (DextNH_2_ )

To 100 mL of amine-dextran polymer solution (10 mg/mL), Mw 1.500 or 6.000, in 5 mM sodium phosphate buffer at pH 7.5 was added 420 µL (0.0625 eq) or 26 (0.0156 eq) µL of a solution 100 mM in acetonitrile of *N*-Succinimidyl-*S*-acetylthioacetate, respectively. The reaction was maintained for 2 h at 25 °C and finally the mixture was extensively dialyzed. Finally, a solution of 100 mM hydroxylamine chloride in 10 mM sodium acetate buffer pH 5.5 was added to the polymers for 30 min for deacetylation (see Fig. 3).

### Protection of thiol in BTL variants by 2-PDS

Different BTL variants (0.2 g of the immobilized form) were incubated in 2 mL of DTT solution (50 mM in 25 mM sodium phosphate at pH 8) for 30 min to avoid oxidation and permitting the posterior disulfide exchange. After, the reduced biocatalysts were washed with distilled water after the DTT smell disappeared.

Then 0.2 g of reduced BTL variants was added to 3 mL of 2-PDS solution (1.5 mM substrate in a mixture of DMSO (5%, v/v) in 25 mM phosphate buffer at pH 8.0) for 1 h. The cysteine PDS activation was followed spectrophotometrically by measuring the increase of the absorbance at 343 nm (by the release of 2-mercaptopyridine, which quickly tautomerizes into 2-thiopyridone) of the solution. A full modification was found in all BTL variants (Godoy et al., 2010).

### Chemical incorporation of tailor-made polymers on different BTL variants

0.7 mL of respective polymer solution was dissolved in 2.3 mL of 500 mM sodium phosphate buffer with 0.5% Triton-X100 at pH 8. Then, 0.2 g of immobilized PDS-BTL variants was added. After 1 h, the modification was confirmed spectrophotometrically by measuring the increase of the absorbance at 343 nm. The biocatalyst was filtered.

### Enzymatic hydrolysis of different substrates

#### Enzymatic hydrolysis of 3’,5’-di-O-acetylthymidine (**1**)

Substrate **1** (5 mM) was dissolved in a mixture of acetonitrile (5%, v/v) in 10 mM sodium phosphate at pH 7.0 or 10 mM sodium acetate at pH 5.0. 0.2 g of biocatalyst was added to 2 mL of this solution at 25 °C. During the reaction, the temperature and the pH value was maintained constant using a pH-stat Mettler Toledo DL50 graphic. The degree of hydrolysis was analyzed by reverse phase HPLC (Spectra Physic SP 100 coupled with an UV detector Spectra Physic SP 8450). For these assays a Kromasil C18 5 µm ϕ (25 cm × 0.4 cm) column was used and the following gradient program (A: mixture of acetonitrile (10%, v/v) in 10 mM ammonium phosphate at pH 4.2; B: mixture of miliQ water (10%, v/v) in acetonitrile; method: 0–6 min 100% A, 6–14 min 85% A–15% B, 14–22 min 100% A, flow: 1.0 mL min^−1^ ). UV detection was performed at 260 nm. The unit of enzymatic activity was defined as micromoles of substrate hydrolyzed per minute per mg of immobilized protein. The monodeprotected 5-OH (**4**) and 3-OH (**5**) were used as pure standards. The retention time was 2.4 min for Thymidine, 9.4 min for **5** and 10.2 min **4** and 19 min for **1**.

#### Enzymatic hydrolysis of dimethyl-3-phenylglutarate (**2**)

Substrate **5** (0.5 mM) was dissolved in 10 mM sodium phosphate or 10 mM sodium acetate at pH 7.0 and 5.0, respectively. Then 0.25 g of immobilized preparation was added to 5 mL of this solution at 25 °C. During the reaction, the temperature and pH value were kept constant using a pH-stat Mettler Toledo DL50 graphic. The degree of hydrolysis was analysed by reverse phase HPLC (Spectra Physic SP 100 coupled with an UV detector Spectra Physic SP 8450). For these assays a Kromasil C8 5 µm (25 cm × 0.4 cm) column was used and the mobile phase was acetonitrile (35%) in 10 mM ammonium phosphate at a final pH value of 3.0. UV detection was performed at 225 nm. The unit of enzymatic activity was defined as micromoles of substrate hydrolyzed per minute per mg of immobilized protein. The retention time was 7.4 min for **6** and 22 min for **2**.

*Determination of enantiomeric excess*

The enantiomeric excess (ee) of the released monoester **6** was analyzed by chiral reverse phase HPLC. The column was a Chiracel OD-R, and the mobile phase was an isocratic mixture of acetonitrile (20%, v/v) in 10 mM ammonium phosphate at pH 3. The detection of the compounds was performed at 210 nm. The retention time was 58.7 min for (*R*)-**6** and 63.2 min for (*S*)-**6**.

### Irreversible inactivation of BTL immobilized preparations by diethyl-p-nitrophenylphosphate (D-pNP)

0.2 g of different BTL immobilized preparations were suspended in 4 mL of 25 mM sodium phosphate buffer solution at pH 7 and 25 °C with or without the presence of 0.5% of Triton X-100. Then, 1.5 mM of inhibitor (D-pNP) was added to this solution. The reaction was maintained until the activity – measured using pNPB assay – of the immobilized enzyme was zero.

## Results and discussion

### Mutagenesis, purification and immobilization of BTL variants

**Figure 4:**
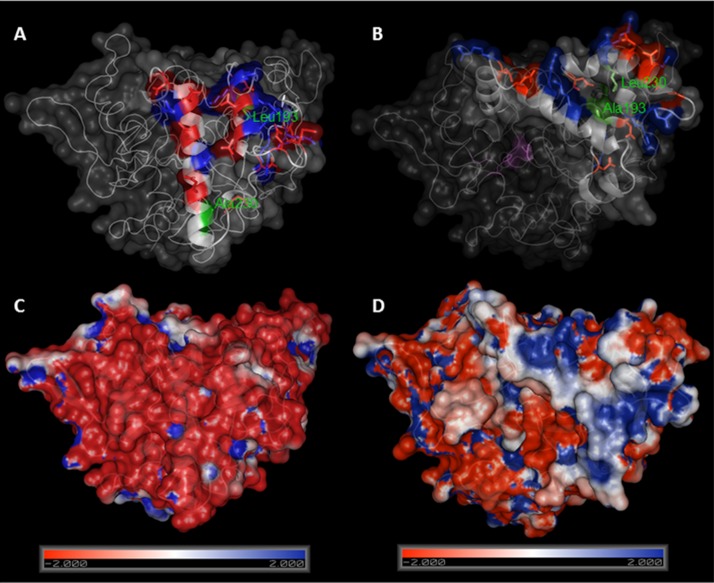
Comparison between crystal structures of BTL in closed (A) and open (B) conformation. Ala193 and Leu230 are marked in green, charged aminoacids in lid zone are highlighted in red (negative) and blue (positive). Electrostatic potential surface representations of crystal structures of BTL in closed (C) and open (D) conformation. Figure was drawn using Pymol 0.99 program and pdb codes: 1KU0 (closed) and 2W22 (open). Electrostatic potential surface was calculated using APBS software.

Lipase BTL has the particularity that its open configuration involves a large structural rearrangement, and the concerted movements of a tricky lid formed by two different loops. We therefore set out to create semisynthetic BTL variants with only one cysteine located at lid zone. Based on a bioinformatics study using the crystallographic open conformation of the enzyme (Carrasco-López et al., 2009) and the charged amino acid distribution in the lid (Baker et al., 2001), two different positions were selected to be mutated: Ala193 (in the internal loop) and Leu230 (in the external loop), see Fig. 4.

First, focused on the protein engineering of this lipase, the two cysteines (Cys65, Cys296) in the wild type enzyme (BTL-WT) were replaced by two serines. The new-engineered enzyme (BTL-C65S/C296S: BTL*) was expressed in *E. coli* without detriment to the enzyme activity. Therefore, using BTL C65S/C296S as template, residues Ala193 and Leu230 were replaced by cysteine residues by directed mutagenesis to create the two single cysteine variants: BTL*-A193C and BTL*-L230C. Both variants were expressed in *E. coli* with a good production similar than BTL-WT and BTL*.

All BTL variants were efficiently purified by hydrophobic chromatography (Fernandez-Lorente et al., 2008) and immobilized on CNBr-activated Sepharose (>95% immobilization yields) with a final loading of 5 mg_lip_/g_cat_ .

### Site-directed polymers incorporations

**Figure 5:**
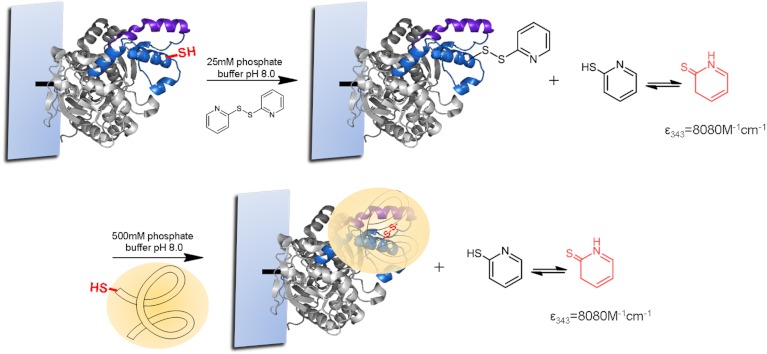
Preparation of site-directed chemical modified BTL catalysts.

For the strategic site-directed chemical incorporation of polymers on lid zone of lipase, the single cysteine variants, already immobilized on CNBr-agarose support, were protected using 2,2-dithiodipyridine disulfide (2-PDS) to allow the disulphide exchange modification and avoiding undesired oxidation (Fig. 5). First, the immobilized variants of BTL, previously reduced with DTT, were incubated in a solution of 1.5 mM 2-PDS at pH 8.0. Then, the modification was followed spectrophotometrically at 343 nm and obtained a modification yield over 95%.

Finally, the different protected BTL variants were specifically modified with a set of tailor-made thiolated-ionic-polymers. The incorporation of the thiolated-ionic-polymer was followed spectrophotometrically by the release of 2-thiopyridone (as in the previous step) (Fig. 5). After 1 h of incubation, a quantitative yield of modification was achieved in all cases.

The BTL* (variant without cysteines) was subjected to a similar treatment in order to confirm that the chemical modification only involved the thiol group. This mutated BTL could not be modified at all by any of these polymers (data not shown).

### Effect of the chemical modification on activity and hyperactivation

The effect of the modification of immobilized BTL* variants using different polymers (different sort and sizes) on the specific activity towards *p*-nitrophenyl butyrate (**3**) is shown in Table 1.

**Table 1:** Activity of the modified biocatalysts in the hydrolysis of pNPB in the absence or in the presence of detergent.

Biocatalyst	Modification	Specific activity^a^	
		without detergent	with detergent^b^
BTL-WT	-	26.5 ± 0.6	65.0 ± 0.9
BTL*	-	27.4 ± 0.5	68.9 ± 1.8
BTL*-A193C	-	26.0 ± 0.3	53.5 ± 0.6
BTL*-A193C	Dext-COOH 1500	20.8 ± 0.6	33.0 ± 0.8
BTL*-A193C	PEI 1500	31.4 ± 0.8	47.0 ± 0.8
BTL*-A193C	Dext-COOH 6.000	13.2 ± 0.3	26.5 ± 0.5
BTL*-A193C	Dext-NH2 6.000	10.6 ± 0.3	23.4 ± 0.3
BTL*-L230C	-	23.0 ± 0.5	45.7 ± 0.7
BTL*-L230C	Dext-COOH 1500	26.0 ± 0.2	71.2 ± 1.4
BTL*-L230C	PEI 1500	22.5 ± 0.5	77.0 ± 1.4
BTL*-L230C	Dext-COOH 6.000	43.0 ± 0.8	125.9 ± 2.3
BTL*-L230C	Dext-NH2 6.000	30.1 ± 0.6	115.8 ± 1.7

**Notes.**

a Specific activity was defined as: µmol min^−1^ mg_prot_ .

b Activity assay in present of 0.5% (w/v) of Triton-X100.

The BTL*-A193C variant maintained the same specific activity than BTL-WT and BTL* variants. The modification of BTL*-A193C with Dext-COOH 1500 caused a slight increase on the specific activity (up to 118%). The enzyme modification with others polymers decreased considerably the activity (from 78% to 40%).

In the case of BLT*-L230C variant, the mutation produced a slight decrease in the activity, but the polymer incorporation improved the activity value in all cases, reaching 162% with Dext-COOH 6000.

The modification of the BTL variants with Dext-COOH 20000 greatly decreased the specific activity of the lipase in all cases (data not shown), this might be caused by steric hindrances with the incorporation of a “big size” polymer.

In order to investigate the effect of the polymer incorporation on the interfacial activation of the lipase, the specific activity in the hydrolysis of **3** was measured in the presence of 0.5% triton-X100 (v/v) (Table 1). As previously reported (Godoy et al., 2011), BTL-WT and BTL* are hyperactivated around 2.5 fold in presence of triton-X100, due to the stabilization of the open form of lipase molecule. Both mono-cysteine variants presented a similar hyperactivation (2 fold).

The hyper-activation showed by the polymer-modified BTL*-A193C variant was similar or even lower than non-modified enzyme. However, this site-directed chemical modification of the BTL*-L230C lipase variant caused a higher hyperactivation of the enzyme in the presence of detergent. In particular, BTL*-L230C-DextNH_2_6000 showed the highest hyperactivation (approx. 4-fold) (Table 1).

These results show, activity and hyperactivation, that the specific incorporation of ionic polymers on the lid area alter the natural closed-open lid movement and maybe this effect is caused by steric hindrances, or the generation of a different lid conformational restructuration.

### Effect of the chemical modification on the irreversible inhibition with D-pNP

**Figure 6:**
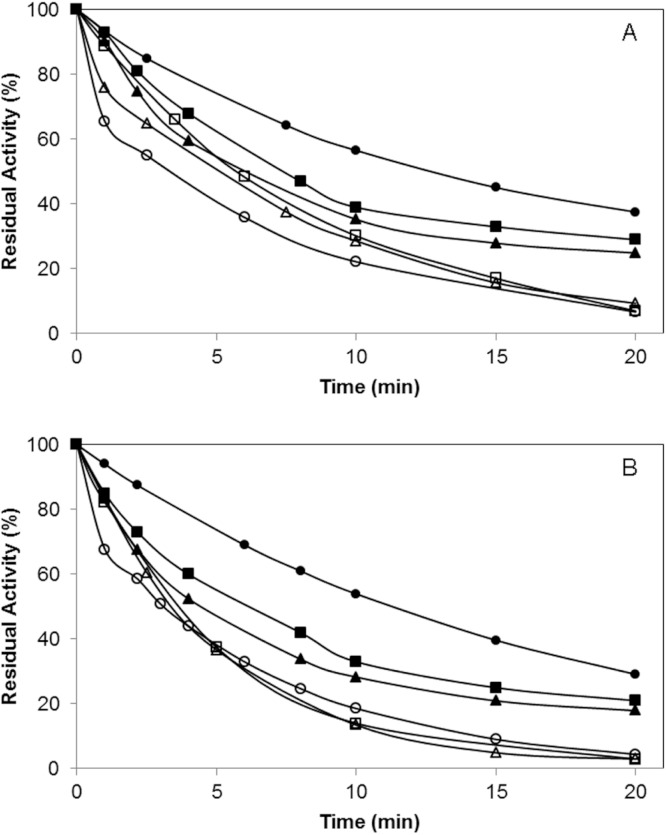
Irreversible inhibition of different biocatalysts using D-pNP.

In order to evaluate the effect of the polymer incorporation of the lipase surface on the enzyme catalytic mechanism, the irreversible inhibition of the enzyme by using diethyl-p-nitrophenylphosphate (D-pNP) was studied. A faster decrease on the specific activity of the enzyme variants was observed after the chemical incorporation of the polymers (Fig. 6).

In the case of the BTL*-A193C variant, after 10 min, this mutated enzyme preserved 57% of the initial activity while BTL*-A193C-DextNH_2_6000 maintained only 29% of activity. When the inhibition was performance in the presence of 0.5% Triton (condition where the conformational equilibrium of the lipase is displaced to the open form), BTL*-A193C conserved the 22% of activity at the same time (Fig. 6A). For the variant BTL*-L230C similar results were observed (Fig. 6B). This result might involve that a more open conformation structure of the lipase was favored in both cases after the polymer modification. Moreover, the size of the polymer also had influence on the final protein structure. The lipase modification with higher molecular size (6000 kDa) polymers caused a faster inhibition than the smaller ones (1500 kDa).

Therefore, it seems that the chemical modification is favouring a higher exposition of the catalytic Ser to the medium, possibly caused by different structural changes in the lid region. It is very likely that it favours an open form of the enzyme with changes in the catalytic properties (Godoy et al., 2010; Romero et al., 2012).

### Biotechnological applications of BTL modified variants

The biotechnological potential of these semisynthetic enzymes has been analyzed in two interesting biotransformations: the regioselective deprotection of per-*O*-acetylated thymidine (**1**) and the desymmetrization of dimethylphenylglutaric acid diester (**2**).

**Table 2:** Regioselective deprotection of 3,5-diacetylated thymidine with different biocatalysts at pH 7.0.

Biocatalyst	Modification	Activity^a^	Time	Yield^b^		
			(h)	**4** (%)	**5** (%)	**B**^c^ (%)
BTL-WT	-	0.55 ± 0.008	80	59	8	33
BTL*	-	0.65 ± 0.007	70	51	5	44
BTL*-A193C	-	0.64 ± 0.008	93	79	8	13
BTL*-A193C	Dext-COOH 1.500	0.59 ± 0.008	93	75	7	18
BTL*-A193C	Dext-NH_2_ 1.500	0.66 ± 0.011	93	78	6	16
BTL*-A193C	Dext-COOH 6.000	1.38 ± 0.019	93	72	3	25
BTL*-A193C	Dext-NH2 6.000	1.64 ± 0.015	93	89	2	9
BTL*-L230C	-	0.77 ± 0.008	57	62	13	25
BTL*-L230C	Dext-COOH 1.500	0.70 ± 0.008	48	72	10	18
BTL*-L230C	Dext-NH_2_ 1.500	0.49 ± 0.007	72	37	16	47
BTL*-L230C	Dext-COOH 6.000	0.15 ± 0.003	144	52	23	25
BTL*-L230C	Dext-NH2 6.000	0.26 ± 0.003	132	76	15	9

**Notes.**

a Specific activity was defined as: µmol min^−1^ g × 10^−5^ .

b Yield of the corresponding product at 100% conversion.

c B: Bihydrolyzed product (thymidine).

First, the BTL variants were tested in the hydrolysis of **1** at pH 7.0 (Table 2). Both mutated variants (BTL*-A193C and BTL*-L230C) showed a slight increase on activity and regioslectivity than the BTL-WT and BTL* toward the production of 3’-*O*-acetylthymidine (**4**). The incorporation of DextCOOH-6000 and DextNH_2_-6000 in the BTL*-A193C increased the lipase activity in 2.5 and 3 fold, respectively. Furthermore, the BTL*-A193C modified with DextNH_2_6000 exhibited a significant increase in the regioselectivity, reaching a yield of 89% of (**4**). However, the modification at the mutated Cys230 did not produce better results, in both activity and regioselectivity. The BTL*-L230C-DextCOOH-6000 variant showed a decrease in activity in more than 3.5 fold and BTL*-L230C-DextNH_2_-1500 exhibited the lowest regioselectivity value (37% of **4**).

In order to investigate the importance of the ionization state of the new biocatalysts, the pH of the reaction was decrease (from 7 to 5), showing significant differences (Table 3). The BTL-WT and BTL* showed around 30% less activity at pH 5.0 than at pH 7.0, but a slightly higher regioselectivity. The most interesting changes were obtained in the BTL*-L230C variant. The specific incorporation of DextCOOH-1500 and DextNH_2_-1500 produced an increase on the activity in 4.5 and 6.0 fold, more than BTL-WT. These new biocatalysts showed better regioselectivity, 84 and 80% yield of (**4**), respectively.

**Table 3:** Regioselective deprotection of 3,5-diacetylated thymidine with different biocatalysts at pH 5.0.

Biocatalyst	Modification	Activity^a^	Time	Yield^b^		
			(h)	**4** (%)	**5** (%)	**B**^c^ (%)
BTL-WT	-	0.37 ± 0.004	96	65	6	29
BTL*	-	0.43 ± 0.006	80	70	4	26
BTL*-A193C	-	0.77 ± 0.010	72	91	3	6
BTL*-A193C	Dext-COOH 1.500	0.86 ± 0.010	72	89	4	7
BTL*-A193C	Dext-NH_2_ 1.500	0.80 ± 0.009	72	90	3	7
BTL*-A193C	Dext-COOH 6.000	1.04 ± 0.014	58	92	3	5
BTL*-A193C	Dext-NH2 6.000	0.85 ± 0.010	72	92	4	4
BTL*-L230C	-	0.55 ± 0.006	69	67	8	25
BTL*-L230C	Dext-COOH 1500	1.78 ± 0.007	55	84	5	11
BTL*-L230C	Dext-NH_2_ 1.500	2.32 ± 0.009	55	80	5	15
BTL*-L230C	Dext-COOH 6.000	0.07 ± 0.001	216	74	19	7
BTL*-L230C	Dext-NH2 6.000	0.11 ± 0.002	264	76	16	8

**Notes.**

a Specific activity was defined as: µmol min^−1^ g × 10^−5^ .

b Yield of the corresponding product at 100% conversion.

c B: Bihydrolyzed product (thymidine).

The BTL*-A193C variant exhibited the higher regioselectivity value at pH 5.0, with 91% yield of **4**. The modification with DextCOOH-6000 increased the activity in nearly 35% without decreasing in the regioselectivity (for time reaction course see Fig. S1).

The desymmetrization of dimethyl phenylglutaric acid diester (**2**) at pH 7.0 and 5.0 was studied after. The two monocysteine-BTL* variants showed higher enantiomeric excess than BTL-WT and BTL* (Tables 4 and 5).

**Table 4:** Asymmetric hydrolysis of dimethyl 3-phenylglutarate with different biocatalysts at pH 7.0.

Biocatalyst	Modification	Activity^a^	Time	Conversion	Yield^b^	e.e^c^
			(h)	(%)	(%)	(%)
BTL-WT		8.48 ± 0.10	196	48	42	68
BTL*		7.61 ± 0.16	196	28	23	64
BTL*-A193C	-	0.53 ± 0.01	186	23	15	78
BTL*-A193C	Dext-COOH 1500	0.48 ± 0.01	186	12	1	n.d^d^
BTL*-A193C	Dext-NH_2_ 1.500	0.59 ± 0.01	186	26	17	87
BTL*-A193C	Dext-COOH 6.000	1.86 ± 0.05	96	43	18	60
BTL*-A193C	Dext-NH2 6.000	2.76 ± 0.02	96	64	20	>99
BTL*-L230C	-	11.33 ± 0.12	96	65	38	93
BTL*-L230C	Dext-COOH 1500	3.41 ± 0.06	72	49	16	93
BTL*-L230C	Dext-NH_2_ 1.500	1.98 ± 0.03	63	29	10	94
BTL*-L230C	Dext-COOH 6.000	1.43 ± 0.02	96	33	17	57
BTL*-L230C	Dext-NH2 6.000	0.99 ± 0.01	96	23	18	92

**Notes.**

a Specific activity was defined as: µmol min^−1^ mg_prot_ × 10^−3^ .

b Yield of monoester **6**.

c Determined by HPLC.

d Not determined.

**Table 5:** Asymmetric hydrolysis of dimethyl 3-phenylglutarate with different biocatalysts at pH 5.0.

Biocatalyst	Modification	Activity^a^	Time	Conversion	Yield^b^	e.e^c^
			(h)	(%)	(%)	(%)
BTL-WT	-	6.77 ± 0.14	196	32	29	72
BTL*	-	4.88 ± 0.10	196	23	21	82
BTL*-A193C	-	2.74 ± 0.05	48	32	26	82
BTL*-A193C	Dext-COOH 1.500	2.22 ± 0.02	48	26	26	45
BTL*-A193C	Dext-NH_2_ 1.500	2.88 ± 0.05	48	33	25	75
BTL*-A193C	Dext-COOH 6.000	0.74 ± 0.01	96	17	7	87
BTL*-A193C	Dext-NH2 6.000	0.81 ± 0.01	96	19	13	96
BTL*-L230C	-	13.49 ± 0.14	96	62	12	96
BTL*-L230C	Dext-COOH 1.500	5.47 ± 0.09	72	85	67	94
BTL*-L230C	Dext-NH_2_ 1.500	6.20 ± 0.09	146	36	27	96
BTL*-L230C	Dext-COOH 6.000	0.37 ± 0.01	192	18	8	79
BTL*-L230C	Dext-NH2 6.000	0.30 ± 0.01	192	14	8	77

**Notes.**

a Specific activity was defined as: µmol min^−1^ g × 10^−3^ .

b Yield of monoester **6**.

c Determined by HPLC.

In the hydrolysis of **2** at pH 7, the activity of BTL*-A193C decreased considerably compared with BTL-WT (more than 17 fold) (Table 4). The incorporation of tailor-made polymers improved the activity and the enantioselectivity. When the BTL*-A193C variant was modified with DextCOOH_2_-6000 the activity increased 3.5 fold. The modification with DextNH_2_-1500 gave a higher enantiomer excess (87%). Moreover, the best result was found after the chemical modification with DextNH_2_-6000. The initial enzymatic activity increased more than 5 fold after this modification and this variant catalyzed the hydrolytic reaction with excellent enantiomeric excess (>99%) (for time reaction course see Fig. S2).

The incorporation of the cysteine in 230 generated a higher active and selective enzyme variant (BTL*-L230C) in the hydrolysis of (**2**) (93% e.e.) (Table 4). However, any modification improved this result, the modified BTL*-230 catalyst with DextCOOH-1500, DextNH_2_-1500 and DextNH_2_-6.000 has a similar enantioselectivity but lower activity. With the performance of BTL*-230, the reaction at pH 5.0, involved a slight increase of both activity and enantioselectivity around 20% and reaching 96% e.e., respectively (Table 5).

## Conclusion

Site-directed incorporation of tailor-made ionic-polymers on the lid-site of two different cysteine-BTL variants -based on a fast thiol-disulfide exchange ligation- was successfully performed. Using different polymers (different sort and sizes), a small library of new biocatalysts was created. This generated biocatalysts showed different and important changes in the catalytic properties, possibly caused by structural changes in the lid region.

The modification of an enzyme variant with specific polymers have permitted the obtainment of biocatalysts with enhanced activity towards non-natural substrates and even excellent enantio- and regioselectivities in the production of two important intermediates in the synthesis of enantiomerically pure drugs. This strategy therefore represents a very rapid, simple and efficient methodology to create biocatalysts with excellent properties for pharmaceutical industrial purpose.

**Abbreviations**
BTL: *Geobacillus Thermocatenulatus* lipase
CNBr: Sepharose^®^ 4BCL activated with cyanogen bromide
D-pNP: diethyl-p-nitrophenylphosphate
DTT: dithiothreitol
EDC: 1-ethyl-3-(3-dimethylaminopropyl)-carbodiimide
NHS: *N*-hydroxysuccinimide
EDA: ethylenediamine
2-PDS: 2,2’-dipyridyldisulfide
BTL-WT: BTL lipase wild type
BTL*: BTL-C65S/C296S lipase mutant
BTL*-193: BTL-C65S/C296S/A193C lipase mutant
BTL*-230: BTL-C65S/C296S/L2293C lipase mutant
Dext-COOH 1500: thiol-aspartic-dextran polymers Mw 1500
Dext-COOH 6000: thiol-aspartic-dextran polymers Mw 6000
Dext-NH_2_ 1500: thiol-amine-dextran polymers Mw 1500
Dext-NH_2_ 6000: thiol-amine-dextran polymers Mw 6000

## Supplemental Information

10.7717/peerj.27/supp-1 Supplemental Information 1 Supporting information.
