# Supplementary material for: Novel enzyme-polymer conjugates for biotechnological applications
Source: PeerJ. 2013 Feb 12;1:e27. doi: 10.7717/peerj.27 (PMC3628993; doi:10.7717/peerj.27)
Supplement: Supplemental Information 1 [file peerj-01-27-s001.docx]

**Supporting Information**

## Table S1. Primers used to site-directed mutagenesis of BTL.

| **Mutant** ^(1)^ | **Plasmid template** | **Primers** ^(2)^ |
| --- | --- | --- |
| C65S/C296S/*A193C* | pT1BTL2mutCys ^(3)^ | Ala/cys 193-5 5´-GAAAGCGtgcGCTGTCGCCAG  Ala/cys 193-5 5´- CTGGCGACAGCgcaCGCTTTC |
| C65S C296S *L230C* | pT1BTL2mutCys | Leu/Cys 230-5 5´-CATTATTTTGAACGGtgcAAACG  Leu/Cys 230-3 5´-CGTTTgcaCCGTTCAAAATAATG |

^(1)^ The mutant name shows the amino acid changes and its position in BTL.

^(2)^ The nucleotide changes used to introduce the mutation are indicated in lower case

^(3)^ Plasmid with BTL mutant lacking both of the two native Cys residues (Cys65 and Cys 296).
